# Total collagen content and distribution is increased in human colon during advancing age

**DOI:** 10.1371/journal.pone.0269689

**Published:** 2022-06-17

**Authors:** Nicholas Baidoo, Ellie Crawley, Charles H. Knowles, Gareth J. Sanger, Abi Belai

**Affiliations:** 1 University of Roehampton, School of Life Sciences, London, United Kingdom; 2 Faculty of Medicine and Dentistry, Blizard Institute, Queen Mary University of London, London, United Kingdom; VIT University, INDIA

## Abstract

**Background:**

The effect of ageing on total collagen content of human colon has been poorly investigated. The aim of this study was to determine if ageing altered total collagen content and distribution in the human colon.

**Methods:**

Macroscopically normal ascending colon was obtained at surgery from cancer patients (n = 31) without diagnosis of diverticular disease or inflammatory bowel disease. Masson’s trichrome and Picrosirius red stains were employed to identify the total collagen content and distribution within the sublayers of the colonic wall for adult (22–60 years; 6 males, 6 females) and elderly (70 – 91years; 6 males, 4 female) patients. A hydroxyproline assay evaluated the total collagen concentration for adult (30–64 years; 9 male, 6 female) and elderly (66–91 years; 8 male, 8 female) patients.

**Key results:**

Histological studies showed that the percentage mean intensity of total collagen staining in the mucosa, submucosa and muscularis externa was, respectively, 14(1.9) %, 74(3.2) % and 12(1.5) % in the adult ascending colon. Compared with the adults, the total collagen fibres content was increased in the submucosa (mean intensity; 163.1 ± 11.1 vs. 124.5 ± 7.8; ***P*** < 0.05) and muscularis externa (42.5 ± 8.0 vs. 20.6 ± 2.8; ***P*** < 0.01) of the elderly patients. There was no change in collagen content of the mucosa. The total collagen concentration was increased in the elderly by 16%. Sex-related differences were not found, and data were combined for analysis.

**Conclusions:**

Greater total collagen content was found in the submucosa and muscularis externa of the elderly human male and female colon. These changes may contribute to a possible loss of function with ageing.

## Introduction

Advanced age is associated with an increased incidence of lower gastrointestinal (GI) disorders [1]. Among these, chronic constipation is more prevalent in the older population (≥ 65years) for both men and women [2], with the incidence rate ranging from 15% - 50% [3]. Faecal incontinence and impaction leading to long term hospitalization have also been reported in the elderly community [4]. These conditions inflict pain, negatively affect quality of life and dignity [5], and significantly increase healthcare costs [6]. Within primary medical care, other bowel disorders frequently reported in the elderly include changes associated with malnutrition, drug- or treatment-induced diarrhoea, diverticulitis, and a decline in the incidence of irritable bowel syndrome [7].

Collagen is the most abundant structural protein and extracellular matrix (ECM) component in mammals [8]. In the human colon, collagen provides structural or architectural matrices for the intestinal cells to juxtapose, thereby creating the biomechanical strength needed to withstand the high intraluminal pressures formed during muscle movements [9]. These include the ability of the colon to retain and propagate semi-solid faecal contents [10,11]. The colonic wall can be divided into four broad functional layers: mucosa, submucosa, muscularis externa (inner circular and outer longitudinal layers), and serosa. Collagen in the mucosa and serosal layer provides little biomechanical contribution, the expandability and strength of the colonic wall relying on the submucosa and muscularis externa [12,13].

The influence of ageing on the organization, relative distribution, and content of collagen within the different functional sublayers of the human colon has been poorly investigated. In one study, increasing age (> 60 years; n = 7) was associated with reduced collagen fibrillar structure and tightly packed fibrils in the submucosa of the descending colon compared to the ascending colon, suggesting region-dependency in how ageing affects collagen structures [14]. Speakman *et al*. [15] reported higher collagen content in internal anal sphincter of ageing human incontinent patients (mean age, 51.5 years), contributing to reduced ability of the anal canal and sphincters to maintain pressure and achieve maximum squeeze pressure, also reported in other [16,17] but not all studies [18,19]. In the aged rat (27-month-old; n = 1), Christensen *et al* [20] described increased collagen content in the left colon using a hydroxyproline assay, accompanied by reduced maximum load strength. However, the authors did not quantify how increasing age might affect the spatial distribution of collagen fibres within the different sublayers of the colonic wall. This is important because collagen in the mucosa, submucosa and the muscularis externa make different contributions to the functions of the colon wall, not reflected by an overall measurement of hydroxyproline content. In addition, caution is required when extrapolating such data from animal studies to humans. This is due to variation in genetic complexities, differences in metabolic rates and life spans affecting rates of ageing, and other confounding factors affecting the terminal bowel and functions associated with species-dependent variations such as diet [21].

We hypothesised that ageing perturbs the distribution of, as well as the total amount of collagen in the human colon. To test this hypothesis on formalin-fixed paraffin-embedded ascending colonic samples, a convenient method of determining the collagen content was developed from established protocols to give better resolution of the distribution of total collagen within the individual functional sublayers.

Analysis of content and morphology of collagen within tissue sections have been determined by tinctorial or immunohistochemical staining. Traditional Masson’s trichrome (MT) and Picrosirius red (PSR) stains are the most routinely used dyes, but do not distinguish between types of collagens [22,23]. Collagen is one of the few proteins containing almost exclusively the amino acid hydroxyproline in most mammals, including humans [24]. Therefore, by quantifying the absolute hydroxyproline as a fixed percentage of the total amino acid, the amount and concentration of collagen can be estimated using a spectrophotometric-based hydroxyproline assay [25]. Our aim was to employ systematic and reproducible methods from these established protocols to assess if ageing impacts the distribution of total collagen content within the mucosa, submucosa and muscularis externa of the human colon and if such changes are dependent on the sex of the individual.

## Materials and methods

### Subject selection

Macroscopically normal ascending colon tissue was obtained as surgical surplus from patients undergoing elective surgery for non-obstructing bowel cancer, following written informed consent. None of the patients who underwent surgery had previous chemoradiotherapy or diagnosis of inflammatory colonic disease or diverticulitis (known to affect collagen structure; [26]). The sections of colon were obtained at least 5–10 cm away from the tumour and were prospectively collected until at least thirty-one patients ranging in age from 22–91 years (17 male, 14 female) were received. This study was approved by the East London ethics committee (REC 10/H0703/71) and the University of Roehampton (LSC 21/339). An overview of patient demographics used in this study is presented in **S1 Fig.**

### Specimen processing

Human colonic tissues (~ 10 x 10 mm) were routinely fixed in 10% neutral buffered formalin and processed in xylene before embedding transversely in paraffin wax (to demonstrate mucosal, submucosal, muscularis externa and serosal layers). Carefully consistent serial-sections at 4-μm-thickness were generated using a rotary microtome (Leica Biosystems, Buffalo Grove, United States) and mounted on super frost-plus glass slides. A minimum of fifty serial sections (into a depth of 200 μm) per sample were cut. The first section of each sample was used for haematoxylin and eosin (H&E) staining. For each histochemical staining, a minimum of eight sections at 16 μm separation per patient were used for total collagen content analysis. To blind the investigators during analysis to the age of the patients, slides were assigned codes during microtomy. Before staining was performed, sections were deparaffinised, rehydrated, and stained for routine H&E, Masson’s trichrome or Picrosirius red.

### Haematoxylin and eosin staining

Tissue sections were manually stained in Harris haematoxylin, differentiated in 0.5% acid-alcohol and counterstained with eosin [27]. The sections were then dehydrated in graded series of alcohol and cleared in xylene. Stained sections were then mounted with Pertex (Sakura) and coverslipped with glass slide (Sakura, Tokyo-Japan). Nuclei were stained dark blue, and the cytoplasmic regions stained red to pink or orange colouration. On screening the H&E sections, none of the stained sections had significant inflammation, tumour, or structural abnormalities [28].

### Masson’s trichrome (MT) staining

All sections were loaded on Artisan Link auto Stainer (Sakura, Tokyo-Japan) for staining according to a slightly modified protocol [29]. Stained sections were then mounted with Pertex and coverslipped with glass slide (Sakura, Tokyo-Japan). Stained components of the colon sections yielded: black for nuclei; red for cytoplasm, muscle, and erythrocytes; and blue for collagen.

### Picrosirius red (PSR) staining

Picrosirius red staining was manually performed based on a slightly modified technique [30]. Sections were stained in PSR solution (0.1% of Sirius red in saturated aqueous picric acid) for 1hr. The sections were washed in two changes of acidified water (0.5% acetic acid) for 2min each, air dried and dehydrated in three changes of 100% ethanol. Stained sections were then cleared in histoclear and mounted with Pertex and coverslipped with glass slide (Sakura, Tokyo-Japan). In bright-field microscopy, collagen fibres appeared red on a pale-yellow background.

### Image acquisition

Image acquisition was captured using brightfield microscope equipped with digital camera. Sequential images were captured from the mucosa, submucosa, muscularis externa and a minimum of 90 percent of the acquired images were analysed. All images from sections stained with MT and PSR were acquired with a 4X objective lens (numerical aperture = 0.20; Nikon instruments, Melville, NY) on an Eclipse C*i* microscope with DS-L4 camera (Nikon) under identical conditions with automatic exposure and elimination of white balance [31]. To maintain image integrity and clarity, all acquired digital images were stored in an uncompressed tagged image file format (TIFF) with 24-bit RGB; 23MB and 20.00 x 14.22 inches (2880 x 2048) resolution. Blue and red colourations for MT and PSR stains respectively, indicative of collagen fibres was separated from background colours by specific thresholding values using the image processing program, ImageJ (1.53g version, National Institute of Health [32,33]). Manual thresholding values were set for, respectively, MT and PSR staining: Hue (135–195; 0–10), Saturation (25–255; 20–255) and Brightness (20–255; 15–255) in ImageJ. The mucosa, submucosa and muscularis externa (containing myenteric plexus between the circular and longitudinal muscle layers) were manually circumscribed with a tracing tool. The mean intensity and the area fraction (percentage of positive pixels per selected region of interest (ROI)) were automatically calculated on each ROI per section and averaged. Collagen from the serosa layer was not evaluated. Total collagen content for each layer of the colon was defined as the proportion of positive pixels or gray values within the mucosa, submucosal and muscularis externa. Percentage total collagen content (in male and female adult) for each ROI was calculated as the percentage of gray value of one ROI ∕ ∑ gray value of all the three regions investigated. All values were obtained from a mean of the measures per each ROI. Power analysis was performed based on pilot studies to determine the minimum number of subjects sufficiently able to detect any age-associated total collagen changes within the three investigated sublayers. A prior estimation of sample size determined that 95% statistical power required n = 9 and n = 13 per group respectively for histological and biochemical analysis.

### Procedure for quantifying total collagen contents

**Fig 1:**
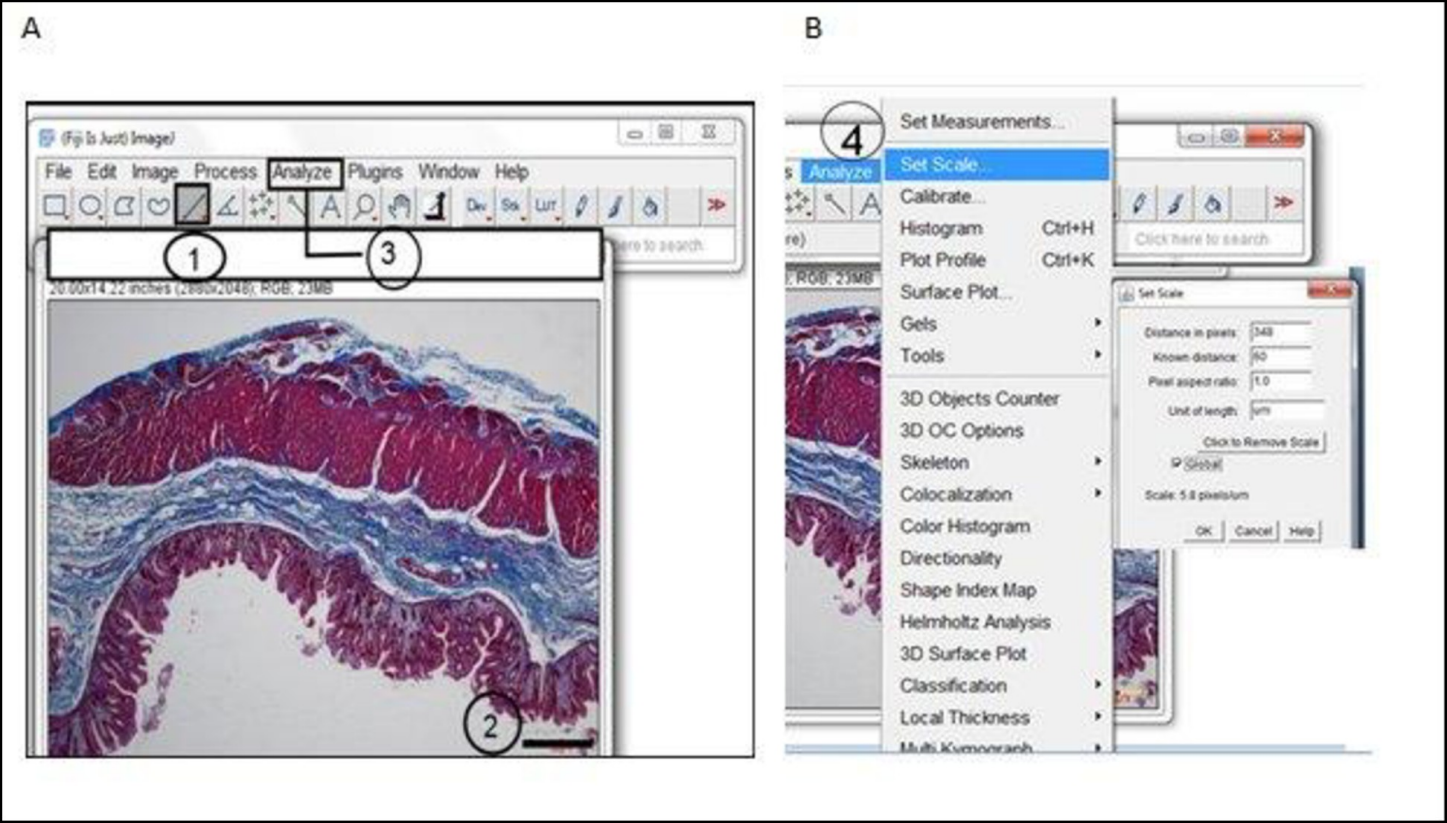
Technique for evaluating colonic samples images with ImageJ programme. The image is first opened, and Scale setting performed by (A): **1**) The “Straight” line tool is selected, and a straight line is drawn as shown on **2.** (B) On the “Analyse” menu (**3**), the “Set Scale” (**4**) automatically calibrates “Distance in pixels”, the length of the scale bar of 60 is entered into “Known Distance” box, and the unit in μm was then input in the “Unit of length” box. And when “Global” is checked, all the images for subsequent analysis were automatically scaled to 5.8 pixels/ μm.

**Fig 2:**
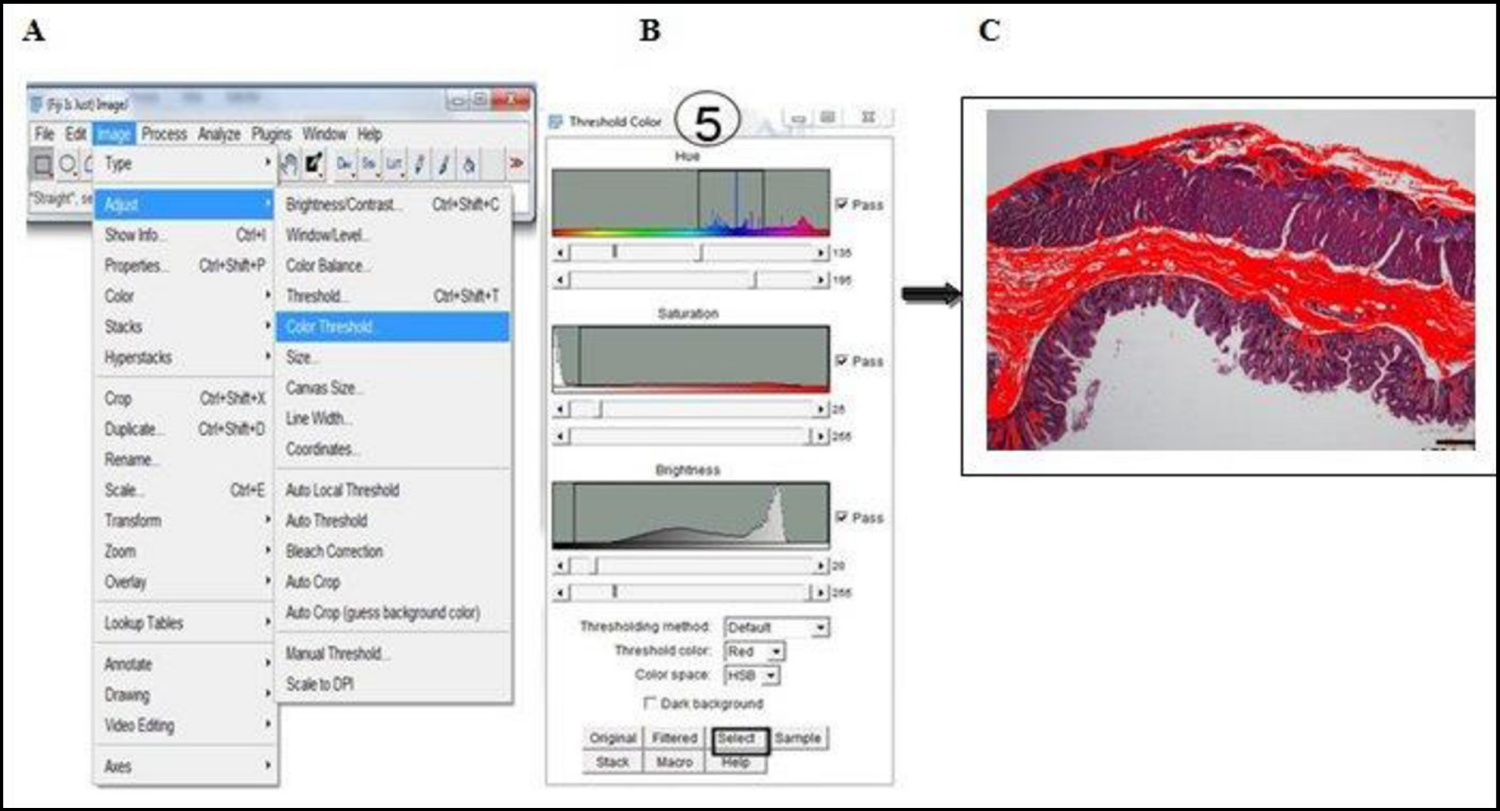
Deconvolution of colour images and identification of blue-stained collagen fibres via colour threshold. A) Under “Image” menu, then to “Adjust” and when “Colour Threshold” is selected, a dialogue box appears (**5**). (**B**) Manual threshold of Hue (135–195), Saturation (25–255) and Brightness (20–255) was set at these values and optimal blue colour intensity was achieved (**C**). Scale bars represent 60 μm.

**Fig 3:**
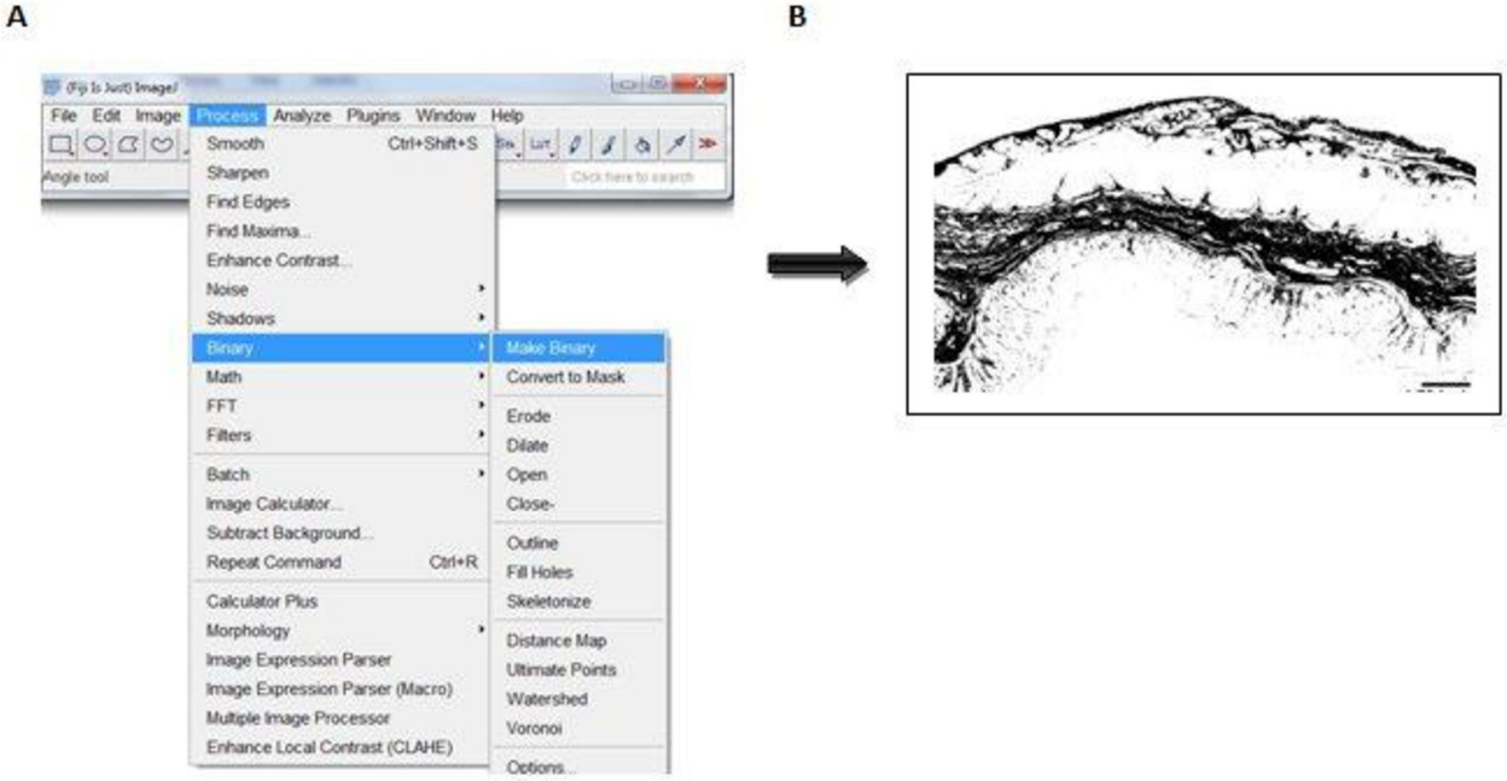
Conversion of threshold image to greyscale for quantification. To convert the threshold image into 8-bit greyscale, (A) “Process” is selected from the menu, and when “Binary” and “Make Binary” is clicked, a greyscale image is achieved (**B**). Scale bar represent 60 μm.

This was performed with a Java-based image processing programme (National Institute of Health, version 1.53g [31,32]). Five steps are required: installation of the ImageJ software and plugin, setting the scale or spatial calibration, deconvolution of the colour images by ‘Manual colour Threshold,’ binarization of image, circumscribing the individual ROI (mucosa, submucosa and muscularis externa) and measurement of positive pixel (**Figs 1–3**). Damaged tissue or insufficient areas in the region of interest were not included in the study.

### Hydroxyproline analysis of collagen concentration

Hydroxyproline concentration in formalin-fixed paraffin-embedded human colonic tissues was measured using an assay kit (*QuickZyme* Biosciences, Netherlands) according to a minor modified procedure [34]. This assay recognizes all types of collagen, irrespective of form (e.g., mature, immature, procollagen, degraded collagen, cross-linked collagen). Ten 10μm sections were transferred to Sarstedt tubes. Samples were then divided into two groups. One group of samples were deparaffinised in histoclear and the other group without it. Thereafter, 150 μl of 6M HCl was added and the samples hydrolysed for 20hr at 95°C in a thermoblock. Hydroxyproline standard solutions (6.25–300 μgmL^-1^) and hydrolysate samples were prepared according to manufacturer’s protocol. Both the standard concentration and sample solution were assayed in duplicate and absorbance at 570 nm read on a multiskan ex microplate reader (Thermo Scientific, Singapore). The averaged blank readings were subtracted from the averaged duplicate readings for each standard and sample. A standard curve was prepared by plotting the mean A_570_ (minus blank) of each standard on the y-axis against the collagen content concentration on the x-axis and applying a best-fit linearized curve through the points on the graph. The unknown concentrations of total hydroxyproline in treated and untreated colonic hydrolysates were deduced per volume of HCl used, based on the standard calibration curve.

### Statistical analysis

The total collagen content and distribution within the mucosa, submucosa and muscularis externa were expressed as mean ± SEM. Comparisons between the two histochemical methods for total collagen staining as well as differences in hydroxyproline content between treated and untreated groups were performed using the Wilcoxon signed rank test for related samples. Age-related changes in total collagen content in the mucosa, submucosa and muscularis externa between an adult and the elderly was compared by a two-tailed independent student’s t-test using the Statistical Package for Social Science (IBM Corp. Released 2019. IBM SPSS Statistics for Windows, Version 26.0. Armonk, NY) software. GraphPad prism software (La Jolla, USA) was used to plot graphs. *P* ≤ 0.05 was considered statistically significant. Unless otherwise specified, ***n*** represent the number of patients. All main results are presented with 95% confidence intervals.

## Results

### Quantification of total collagen content by ImageJ analysis

**Fig 4:**
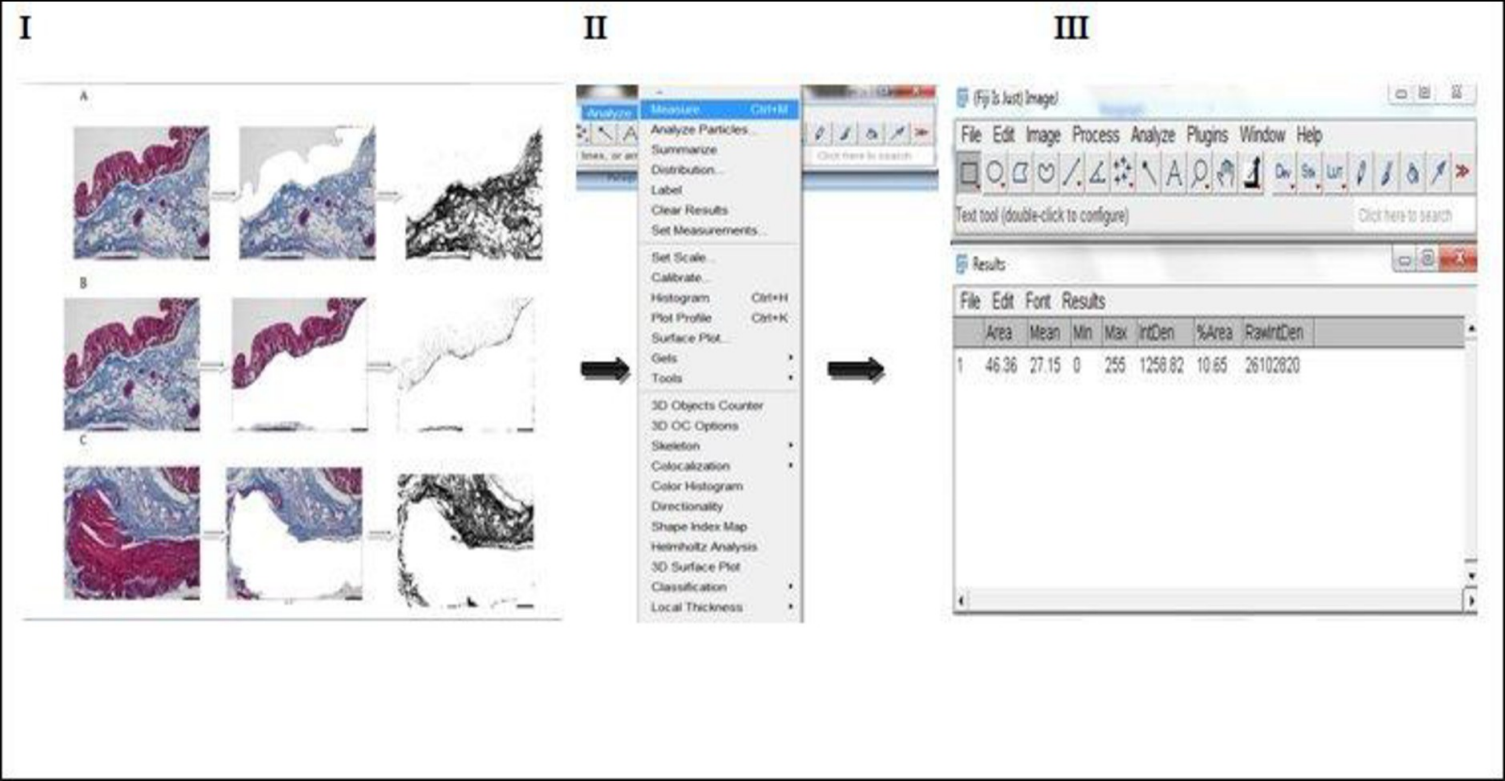
Quantification of total collagen fibres distributed in human colon using ImageJ software. (**I**) Grayscale images of **Masson’s** trichrome stained colonic sections were circumscribed with freehand tool in the (**A**), mucosa (an area consisting of the entire epithelium and muscularis mucosae); (**B**) Submucosa (region below the muscularis mucosae to the area just below the edge of circular muscle layer) and (**C**) Muscularis externa (an area of the circular and longitudinal muscle layer without the serosa). (**II**) When “Analyse” from the menu and “Measure” is selected, the result is presented in (**III**). Scale bars represent 60 μm.

An example process for the described method of analysing the total amount of collagen distribution in the mucosa, submucosa and muscularis externa is shown in **Fig 4.**

### Changes in morphology and distribution of collagen fibres

**Fig 5:**
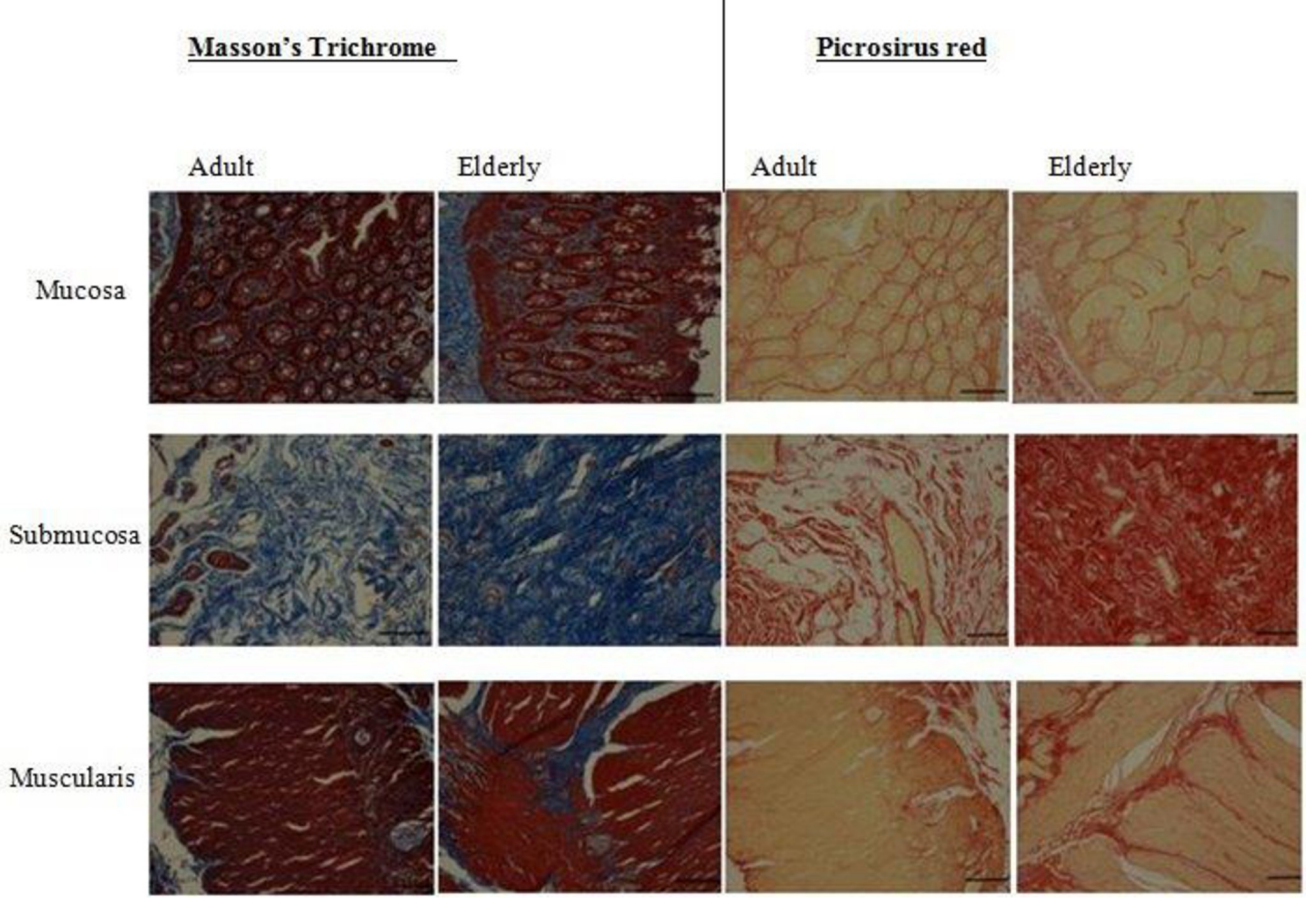
Identification of collagen fibres with histochemical staining. Representative photomicrographs of the mucosa, submucosa and muscularis externa of adult and elderly human colonic tissue showing the distribution pattern of collagen fibres stained with Masson’s trichrome (blue structures indicate collagen) and Picrosirius red (red fibres indicate collagen). Scale bars represent 1mm.

MT and PSR staining labelled all collagen fibres in adult and elderly samples (**Fig 5**). In the mucosa of both groups, collagen was present ensheathing the epithelium and in large part, at the lamina propria.

**Fig 6:**
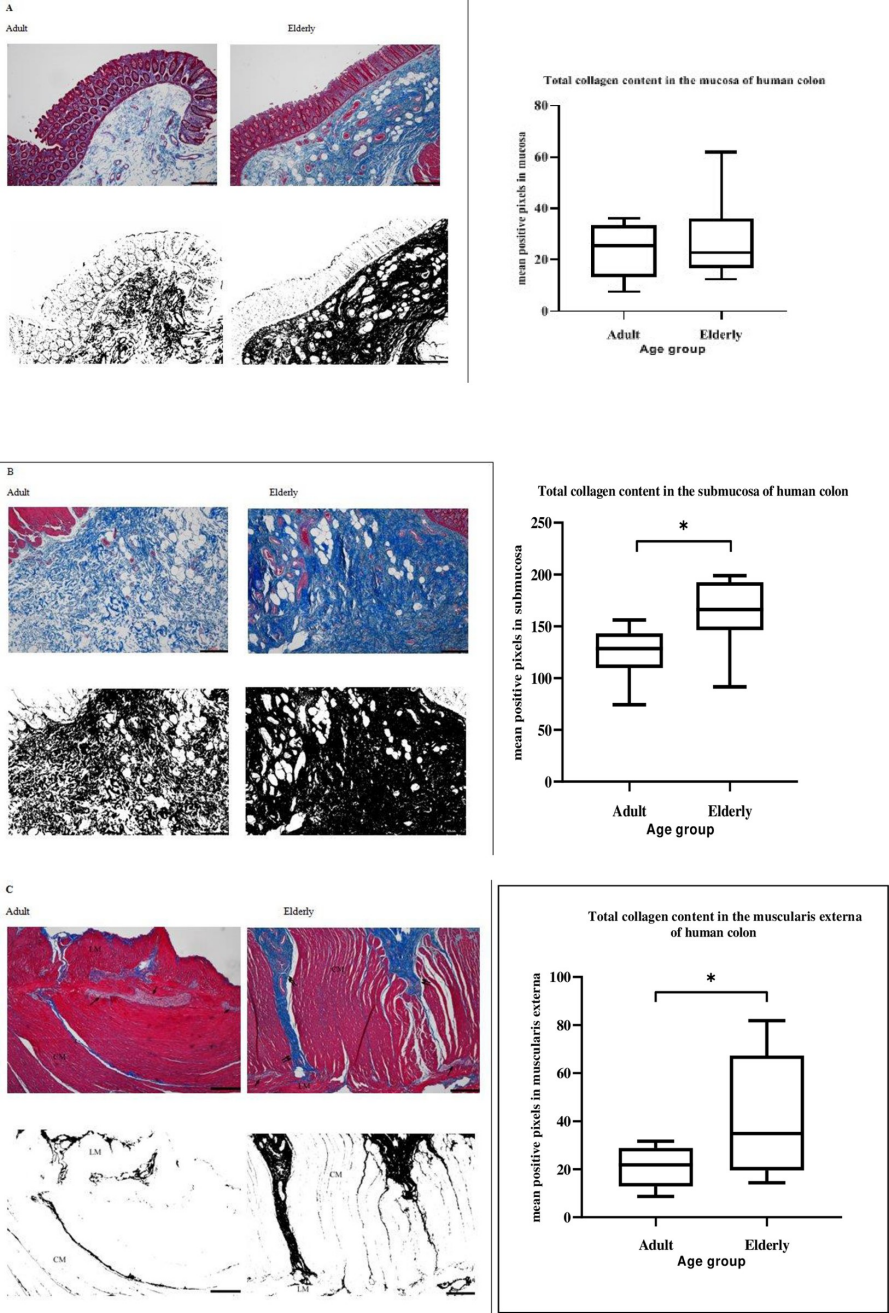
Collagen distribution in the sublayers of human ascending colon. Representative photomicrographs of formalin-fixed paraffin-embedded sections of full-thickness adult and elderly human ascending colon stained with Masson’s trichrome showing the distribution pattern of collagen fibres in (**A**) mucosa, (**B**) submucosa and (**C**) muscularis externa (single arrow: Myenteric plexus; double arrow: Thick collagen fibres; CM and LM: Circular and longitudinal muscle, respectively). Separation of blue colour representing collagen fibres from the background and filtered images converted to black and white using ImageJ processing. Scale bars represent 60 μm. Box plots show the positive pixel of collagen content ± SEM (*n* = 22 patients) distributed within the mucosa, submucosa and muscularis externa between the adult and the elderly as measured by ImageJ. Each ROI were selected with a tracing tool and positive pixel values obtained from binarised image per the selected layer. All data were obtained from a mean of each ROI. Total collagen content between adult and the elderly per each ROI were compared by a two-tailed independent student’s t-test. Statistical significance is: * *P* < 0.05.

When the positive-stained collagen was separated from the background, black oval-shaped structures were visible. Binarized images of the mucosa showed collagen fibres in the mucosa wrapping around individual colonic glands. In the elderly samples, the trajectories of the collagen fibres surrounding the glands were less well-defined, as compared to the adult samples (**Fig 6A**). In the submucosa of both samples, tightly packed collagen fibres of different shapes, sizes and length were identified by both staining methods. In the adult samples, these were seen as a fine network of a wavy organized pattern compared to that of the elderly, thickened and compacted bundles of collagen fibres were seen throughout the submucosa (**Fig 6B)**. In the muscularis externa, MT and PSR staining provided a clear visualization of varying thicknesses of collagen bundles from the submucosa through to the circular muscle to the myenteric ridge ensheathing the myenteric plexus. Small amount of collagen fibres were observed within the taenia coli of the adult samples (See **Fig 6C).**

### Percentage total collagen fibres distribution

Samples from male (n = 6) and female (n = 6) adult patients provided data that overlapped (e.g., mean intensity of mucosal total collagen fibres using MT technique was 22.9 ± 8.3 vs. 24.5 ± 3.8 for male and female adult samples respectively *P* > 0.05). Accordingly, all data were considered together. In some samples, sections with artefacts in a ROI were not included in the quantification. Therefore, where there was a defect in the submucosa for example, the mucosa and the muscularis externa were still analysed from the same patient. Using MT and PSR analysis, the total collagen content distributed in the mucosa, submucosa and muscularis externa were respectively: 14 (1.9) %, 74 (3.2) % and 12 (1.5) %. Total collagen content within the serosa layer was not measured. These values were a mean of the measures of each technique. Although the mean intensity values for PSR staining was high compared to MT (e.g., respectively, mean intensity for adult submucosa: 121.2 ± 7.3 vs. 125.9 ± 10.2 by MT and PSR techniques), indicating different staining sensitivities of collagenous protein across all the ROI investigated, there were no statistically significant differences in the total collagen content in human colon measured by either technique *(Z = -1*.*826*, *P = 0*.*068)*.

### Effect of ageing on the total collagen content distribution

No statistically significant differences of total collagen content were observed in the mucosa between the age groups studied (**See Fig 6A**). Similarly, the total collagen content in the mucosa was unchanged when the percentage area fraction (respectively 10.7% ± 1.8% vs. 9.4% ± 1.3% for adult and elderly; ***P*** = 0.58) was calculated in the adult and the elderly. However, the total collagen fibre content in the submucosa was increased in the elderly compared with adult samples (**See Fig 6B).** Further, the percentage area fraction of total collagen content was higher in the submucosa for the elderly compared with the adult groups (63.9% ± 13.0% vs. 48.8% ± 3.0%; ***P*** < 0.05). In the muscularis externa, advancing age increased the total collagen content (**See Fig 6C)** and percentage area fraction (16.0% ± 3.1% vs. 8.1% ± 1.1%; ***P*** < 0.01) compared to adults.

### Total hydroxyproline concentration

The standard curves fitted between measured absorbance values (A_570_ nm) and standards of known collagen concentration and total converted-hydroxyproline concentration in hydrolysated samples are presented in **S2 Fig.** The hydroxyproline content was higher in the elderly compared to the adult group (**Table 1**; *P* < 0.05). An important concern was whether the use of paraffin-embedded colonic tissue might result in an incomplete acid hydrolysis. However, in additional experiments, similar data were obtained with deparaffinized samples with histoclear (**Table 1**). In relation to total hydroxyproline concentration detection power, there were no significant differences among the measurements obtained in the treated and untreated samples (**Table 1**; *P* > 0.05). There was no difference in the amount of collagen accumulation in the human colon between elderly male (n = 8) and elderly female (n = 8) [(49.9 ± 1.5 vs. 54.8 ± 1.9 *P* > 0.05)].

**Table 1:** Hydroxyproline content in human ascending colon.

		Paraffinized (*n* = 31)	Deparaffinized (*n* = 31)	*p* Value (Methods)
**Adult**		36.9 ± 1.9	37.94 ± 2.1	NS
**Elderly**		51.3 ± 1.7	52.08 ± 1.6	NS
***p* Value Correlation with age**		< 0.05	< 0.05	

Total converted-hydroxyproline concentration expressed as μgml^-1^ per volume of HCl from the formalin-fixed paraffin-embedded (paraffinized and deparaffinized) human ascending colon. The amount of Hydroxyproline concentration was derived via linear coefficient of determination from a standard known concentration. Differences in Hydroxyproline concentration between adult and elderly samples were compared by two-tailed independent test. NS: No significant difference between treated and untreated samples. Significant differences defined by *p* < 0.05. Results are expressed as mean ± SEM.

## Discussion

Ageing has been associated with impaired motility in the terminal regions of the GI tract, implicated in conditions such as constipation, faecal impaction, and incontinence [1]. Recent studies on human colon reported a decline of cholinergic function in ascending but not descending human colon during ageing [35], making this region an interesting area for further investigation.

In this study, histological investigations were performed using human ascending colon to elucidate changes in the amount of total collagen content distributed within the mucosa, submucosa and muscularis externa during ageing. To quantify collagen fibres within an appreciable thickness of colonic samples, the study employed serial sectioning of tissue blocks into a depth of 200 μm. This systematic approach to accurate morphometric investigations using tissue sections has proved useful in other studies [36,37]. The present results confirm that colonic collagen fibres are distributed, aligned and orientated in a layer-dependent manner within the human ascending colon [38,39].

Identification of collagen fibres using MT stain has been widely employed for over a century with either aniline blue or light green dyes [29], and PSR is a highly sensitive detector of connective tissue networks under both brightfield and polarized light [22]. In the present study MT and PSR staining enabled a determination of the total collagen content within the three investigated layers of the human ascending colon. Quantification of histological staining with more than one colour in colonic tissue section is challenging because the main colours often co-localize in the same area within the colon wall. In this study, we used an image processing programme ImageJ which has the capacity to deconvolute colours into 8-bit monochromatic colour thus aiding accurate quantification [32,33]. The blue-stained structures for MT and the red-stained structures for PSR were maximally separated by the “Image Threshold” plugin and a standardisation step during the individual colonic layer quantification was applied to all the samples analysed.

The mucosal collagen content did not differ between adults and the elderly. In adult samples, mucosal collagen fibres were noted to have an oval shaped structure appearing to wrap around individual glands. The circularity of intestinal mucosal collagen is consistent with what has been previously described [38,39]. In contrast, most of the mucosal collagen fibres in the elderly samples appeared to be oriented non-circularly. Whether or not ageing results in a change in the structure and/ or orientation of mucosal collagen fibres, potentially impacting the functional role of the mucosa, must be investigated further. This is important as the differentiation, organisation, architecture and function of the mucosa relies on the result of close functional interactions between the epithelial cells and their underlying collagen fibres within the lamina propria [40].

The current study reported an increase in total collagen fibres in the submucosa of ascending colon of the elderly compared to adult human. In addition, thicker collagen bundles were prominent in the submucosa region of the older samples, mostly towards the edge of the deep circular muscle layer. Since the load-bearing region of the colon is mostly centred in the submucosa [12,13], which contain the submucosal plexus, it may be speculated that thickened collagen bundles in this region would encumber the ability of the neurons to influence the connecting circular muscle activity and the mucosal function. Further, perturbation of submucosal collagen fibres may disrupt the normal function of sensory nerve fibres innervating the mucosa, see [41,42]. Therefore, important submucosal roles including the control of luminal surface, glandular secretions, water transport and local blood flow regulation by these neurons [41–43], are likely to be affected.

The total collagen content was increased in the muscularis externa of the elderly compared to the adult. This has not previously been demonstrated in ageing human colon, although a similar result has been reported in *taenia coli* of 2-year-old guinea pigs compared with younger animals [44]. One of the most important functions of the colon is the ability to temporarily store luminal contents and propagate them for expulsion. Thicker bundles of collagen fibres were noticeably present within the circular muscle of the muscularis externa of the elderly samples. These fibres appeared to extend between the submucous and the deep circular muscle layer and onto the myenteric plexus. Other collagen fibres were also seen to extend between the serosal layer and the *taenia coli* of the elderly. This may be consistent with the higher occurrence of total collagen content in the muscularis externa observed in the elderly samples. Thus, the overall tensile and “burst strength” properties of the muscle would be impaired as reported in a physiological study in ageing rat colon [45]. Together, the present results therefore suggest that age-related changes in total collagen content in the muscularis externa and submucosa may be an important factor in the functional changes described in the elderly ascending colon [35]. A parallel physiological and extracellular matrix studies is warranted to establish this possibility.

Our study is the first to utilise formalin-fixed paraffin-embedded human colonic samples to accurately determine hydroxyproline concentration, although this method has been described over the last three decades using tissue sections from other parts of the body [46]. The increase in total collagen concentration via gold standard hydroxyproline assay affirmed our findings in the histomorphometeric analysis. These data are also consistent with the elevated hydroxyproline content and concentration that was accompanied by a decrease in maximum load strength in the left colon of 27-month-old rat compared to the 4-month-old rat [20]. Quantification of percentage total collagen content confirmed that in both male and female adult colon, the highest total collagen content is found within the submucous layer, potentially serving as the key load-bearing structure in the colon [12,13]. Smaller amounts of collagen were found within the mucosa and muscularis externa. The serosa has a significant content of collagen fibres but is unlikely to have a major load-bearing function [13], so was excluded from this study.

The mechanism (s) which leads to changes in collagen structure and arrangement in the GI tract during ageing are unclear. However, although not analysed in this study, we speculate that thickened collagen fibres observed may have a high degree of cross linkage due to advanced glycation end products (AGEs), carbamylation and fragmentation associated with advanced age [47,48]. An increased accumulation of covalently linked sugar molecules is found on many collagenous proteins and other macromolecules associated with ageing [47]. In this instance, an increased cross linkage of the microstructure samples of the elderly could result in collagen losing its flexibility, becoming stiffer or rigid, susceptibility to enzymatic degradation resistance and loss of solubility property [47–49]. Clearly, this mechanism will have an adverse effect on the biomechanics of the colon. It is established that constipation is one of the most common colonic disorders among the elderly; the prevalence rate appears higher in women than in men [50]. However, the present study suggests that age-related alterations of total collagen deposition within the sublayers of the colon are not based on sex differences.

A weakness of the current study is that it was not possible to use human tissue from healthy individuals. Instead, we examined ‘macroscopically normal’ tissue removed from cancer patients prior to chemotherapy, taken 5–10 cm from the tumour. Although this is a method often used to study the functions of the human gastrointestinal tract [see 51], it does not necessarily eliminate the possibility that the cancer could influence this part of the colon. For example, in the mucosa adjacent to a tumour (up to 10 cm away) in human colon, changes in gene expression [52] and a subtle alteration of collagen fibre organisation have been reported [53]. Nevertheless, by consistently using tissues obtained in this way from bowel cancer patients, age-related changes in gene and protein expression can be identified [54]. In the present study, we found no suggestion of cancer or active inflammation by performing routine haematoxylin and eosin staining, all tissues were removed from cancer patients, the observed changes in collagen content argue for an association with ageing not cancer.

Much remains to be understood on the mechanism of collagen fibres deposition and degradation in human colon during ageing. However, understanding the structural interplay between age-related changes in intestinal collagen properties and the functions of the enteric (neurons and glial cells) and extrinsic nervous systems, of which biomechanical and motility functions are determined, will advance our understanding of age-associated bowel disorders.

## Conclusions

The present study revealed an increase in collagen fibres within the submucosa and muscularis externa of ageing human colon compared to adults. This in turn may contribute to the changes reported in motility function of the colon. Alterations in the amount of collagen fibres and spatial distribution within the three investigated areas can potentially be a marker for colonic disorders commonly reported in older people. Remarkably, age-related changes in the relative distribution of collagen in muscularis externa are more pronounced than the rest of the sublayers of the colon; although differences in collagen content occurrence may exist between the two functional muscle layers which may have a concomitant effect on the resident cells. Differences in the amount of total collagen content in the colonic wall during ageing were not sex dependent. These findings provide an additional perspective into the effect of ageing on the amount of total collagen in the sublayers of human colon.

## Supporting information

S1 Fig Human ascending colonic tissues included in the study. (DOCX)

S2 Fig Hydroxyproline standard curve. The graph demonstrates standard curve of mean absorbance (A570 –blank) against standard known collagen concentration. The standard curve was used to convert the A_570_ values of the test samples to converted-hydroxyproline concentration of collagen in the hydrolysed human colonic samples.
(DOCX)
