# Supplementary material for: Total collagen content and distribution is increased in human colon during advancing age
Source: PLoS One. 2022 Jun 17;17(6):e0269689. doi: 10.1371/journal.pone.0269689 (PMC9205511; doi:10.1371/journal.pone.0269689)
Supplement: S1 Fig — (DOCX) [file pone.0269689.s001.docx]

**Figure S1.**

Human ascending colonic tissues included in the study.

| **Age (y)** | **Sex** | **Diagnosis** | **Comorbidity** | **Medication** |
| --- | --- | --- | --- | --- |
| 22 | M | Cancer | Unknown | Unknown |
| 30 | M | Cancer | Unknown | Unknown |
| 32 | M | Cancer | Unknown | Unknown |
| 47 | F | Cancer | Graves’ disease | Carbimazole |
| 50 | M | Cancer | None | Rampril |
| 51 | M | Cancer | Unknown | Unknown |
| 51 | F | Cancer | Gastritis | Paracetamol |
| 56 | F | Cancer | Unknown | Unknown |
| 57 | M | Cancer | None | Nonregular |
| 58 | F | Cancer | Unknown | Unknown |
| 59 | M | Cancer | Unknown | Unknown |
| 60 | F | Cancer | Diabetes | Nonregular |
| 60 | M | Cancer | Unknown | Unknown |
| 62 | M | Cancer | Unknown | Unknown |
| 64 | F | Cancer | Unknown | Unknown |
| 66 | F | Cancer | None | Folic acid |
| 69 | M | Cancer | None | Amlodipine |
| 70 | M | Cancer | Non-insulin dependent diabetes | Nonregular |
| 71 | F | Cancer | None | HRT, Lansoprazole |
| 75 | M | Cancer | Unknown | Unknown |
| 76 | F | Cancer | None | Clopidogrel |
| 77 | M | Cancer | Gastroduodenitis | Nonregular |
| 77 | F | Cancer | Pancreatic tail lesion | Levothyroxine |
| 80 | F | Cancer | Type 2 Diabetes | Siozem |
| 81 | M | Cancer | Unknown | Unknown |
| 82 | F | Cancer | Bowens disease | Allopurinol |
| 84 | M | Cancer | None | Lipitor |
| 88 | F | Cancer | High blood pressure | Bendroflumethiazide |
| 88 | F | Cancer | Unknown | Unknown |
| 89 | M | Cancer | None | Codeine |
| 91 | M | Cancer | Unknown | Unknown |
