# Supplementary material for: Total collagen content and distribution is increased in human colon during advancing age
Source: PLoS One. 2022 Jun 17;17(6):e0269689. doi: 10.1371/journal.pone.0269689 (PMC9205511; doi:10.1371/journal.pone.0269689)
Supplement: S2 Fig — The graph demonstrates standard curve of mean absorbance (A570 –blank) against standard known collagen concentration. The standard curve was used to convert the A570 values of the test samples to converted-hydroxyproline concentration of collagen in the hydrolysed human colonic samples. (DOCX) [file pone.0269689.s002.docx]

**Figure S2**.

Hydroxyproline standard curve.

The above shown graph demonstrate standard curve of mean absorbance (A_570_ – blank) against standard known collagen concentration. The standard curve was used to convert the A_570_ values of the test samples to converted-hydroxyproline concentration of collagen in the hydrolyzed human colonic samples.
